# Health Care Organization in Poland in Light of the Refugee Crisis Related to the Military Conflict in Ukraine

**DOI:** 10.3390/ijerph20053831

**Published:** 2023-02-21

**Authors:** Artur Prusaczyk, Magdalena Bogdan, Shlomo Vinker, Mariusz Gujski, Paweł Żuk, Iwona Kowalska-Bobko, Sabina Karczmarz, Joanna Oberska, Katarzyna Lewtak

**Affiliations:** 1 Medical and Diagnostic Center, 08-110 Siedlce, Poland; 2 Department of Social Medicine and Public Health, Medical University of Warsaw, 02-007 Warsaw, Poland; 3 Department of Family Medicine, Sackler Faculty of Medicine, Tel Aviv University, Tel Aviv 69978, Israel; 4 Department of Public Health, Medical University of Warsaw, 00-097 Warsaw, Poland; 5 Institute of Public Health, Faculty of Health Science, Jagiellonian University Medical College, 30-688 Krakow, Poland

**Keywords:** health care, refugees, care organization, military conflict, strategy implementation, Ukraine, Poland

## Abstract

Background: Poland is witnessing a migration crisis caused by the ongoing military conflict in Ukraine. In addition to housing and necessities, 1.8 million Ukrainians that had taken refuge in Poland must have access to medical care. We aim to propose a strategy for implementing the changes in the Polish health care system in response to the Ukrainian refugee crisis. Methods: A literature review on organizational changes in the functioning of health care systems during the migration crises worldwide in recent years and brainstorming in order to develop a strategy for implementing changes in the Polish health care system in response to the Ukrainian refugee crisis. Results: The proposed strategy for implementing the changes in the Polish health care system is based on building health care resilience and adaptation to different crises. The operational objectives of organization-related activities are: (1) preparation of medical facilities to provide help for refugees, (2) development and implementation of the communication system, (3) implementation of available digital solutions, (4) organization of the diagnostic and medical services, (5) and implementation of changes in the management of medical facilities. Conclusions: Urgent reorganization is required to respond to an unavoidable increase in the demand for health care services.

## 1. Introduction

Poland is witnessing a migration crisis caused by the ongoing military conflict in Ukraine. By 10 February 2023, more than 9.7 million refugees have crossed Polish border [1]. According to a report conducted among Ukrainian refugees, as many as 69% of them are planning to return to Ukraine as soon as possible, whereas 31% would like to stay in Poland longer, for at least a year (6%), several years (18%), or permanently (7%) [2]. So far, a little over 1.5 million Ukrainian refugees have applied for temporary national protection scheme in Poland [3]. In addition to housing and basic necessities, they must have access to medical care. The increase in population means that the Polish health care system must evolve into a system suitable for a larger and more diverse population.

Access to health services for refugees varies substantially across Europe [4,5,6,7]. Countries with the most favorable legislation for migrants (e.g., Ireland, Sweden, Finland, and Portugal) regarding health care are successfully sharing comprehensive information on entitlements and use of health services with migrants. Interpreters are available and free of charge for patients. Countries are involving migrants in information provision, service design, and delivery. Funding bodies are supporting research on occurrence of health problems among migrants, social determinants of migrant health, issues concerning service provision and evaluation of methods for reducing inequalities in health or health care affecting migrants. Commitment to providing equitable health care for migrants is present in all departments of service provider organizations and health agencies [4,6,8,9]. Some countries (especially those considered first arrival points for migrants in Europe) provide at-border health screening services for infectious diseases, collect data on infections and vaccination, and have regulated surveillance systems [6,10,11,12].

In guidance for health care professionals at border areas, the European Centre for Disease Prevention and Control (ECDC) recommends screening for vaccination status (including vaccination against COVID-19). People with chronic mental health conditions or in need of complex mental health and psychosocial support should be identified and referred to appropriate services. Health care workers should provide information on how to access influenza vaccinations, availability of treatment for HIV and tuberculosis (TB), and contact details of facilities where persons with non-communicable diseases (NCDs) can access care and continuity of treatment and refer to pre-identified clinics and hospitals where people can access advanced care, e.g., for cancer. Special attention should be provided to pregnant women and parents, who should be educated on pregnancy and child health-related matters [13].

The Migrant Integration Policy Index 2020 (MIPEX) measuring policies to integrate migrants in 56 countries across the world placed Poland in second lowest category “Equality on paper—Slightly unfavorable” meaning Polish legislation does not grant migrants equal opportunities in health care access and the information provided on entitlements and health issues is not sufficient [4].

The migration crisis related to the war in Ukraine has highlighted the shortcomings of the health care system in terms of resource availability and capacity. It forced not only temporary organizational and economic changes, but also the need to take them into account in formulating a strategic systemic approach. The Polish health care system lacks a stable, long-term, and predictable vision for its functioning. Public expenditure on health care is relatively low and its allocation is suboptimal [14]. In addition, the health care system faces a shortage of staff (doctors, nurses, and midwives) [14]. There are also problems related to the organization of work, including an old fashioned definition of the roles of individual professionals and an ineffective division of responsibilities between health care workers and between hospitals and community. The COVID-19 pandemic has improved the use of digital technologies by health care entities, but these technologies require more widespread implementation [15]. Additionally, medical, logistic, and translational competences must be skillfully used for the benefit of the refugees.

The characteristics of the refugee population are currently poorly documented. Therefore, any estimation of the impact of the refugee influx on the basis of incomplete data can only result in an approximate description of the resources and services demanded from the Polish health care system. An important element in determining refugee needs is the assessment of the prevalence of communicable and non-communicable diseases in the refugee population. The rate of routine vaccinations among Ukrainian children is far lower than in other European countries, which has even led to local outbreaks of diseases such as polio and measles [16]. Additionally, COVID-19 vaccination rates in Ukraine are much lower than in Poland; 34.8% as opposed to 58.8% [17].

Table 1 presents a description based on the basic data on health care resources and activity indicators in Poland and the estimated need to increase resources and health services as a consequence of the influx from Ukraine into Poland.

The study aims to propose a strategy for implementing the changes in the Polish health system in response to the Ukrainian refugee crisis based on the WHO building blocks framework, including the following elements: (1) service delivery, (2) health workforce, (3) health information systems, (4) access to essential medicines, (5) financing, and (6) leadership/governance, as well as process elements (access, coverage, quality, and safety) and outcomes (improved health and health equity, responsiveness, social and financial risk protection, and improved efficiency) [19].

## 2. Materials and Methods

The study consisted of two main elements: (1) the literature review on organizational changes in the functioning of health care systems during the migration crises that have occurred both in Europe and all over the world in recent years, and (2) brainstorming in order to develop a strategy for implementing changes in the Polish health care system in response to the Ukrainian refugee crisis.

Legal acts that influenced the organizational and economic changes in health care in Poland and were introduced in the period from the outbreak of war in Ukraine, i.e., 24 February 2022, to the end of 2022 were reviewed. This was an important element of the analysis as a background for changes related to health care for migrants and war refugees in Poland. The analysis also includes expert publications affiliated with international organizations on the provision of health care to migrants and war refugees. For the literature review, we undertook a search using the following databases: PubMed, Scopus, and Web of Science. We did not search grey literature sources. The keywords used in the literature search (search strategy) included: resilient health care system, health care system response, health care delivery, migrant*, war refugee*. The inclusion criteria were: (1) articles in English, (2) full-text publications, (3) use of a clear study design (cross-sectional or observational studies, etc.), and (4) studies published in the period 2015–2022 (time of the migration crisis in Europe). Exclusion criteria were non-English articles. We identified 1138 articles via database searches. After excluding duplicates, 878 studies were included in the screening. After screening the titles, n = 88 were considered for further analysis, of which n = 43 were included in the present analysis after reading the full text.

**Figure 1:**
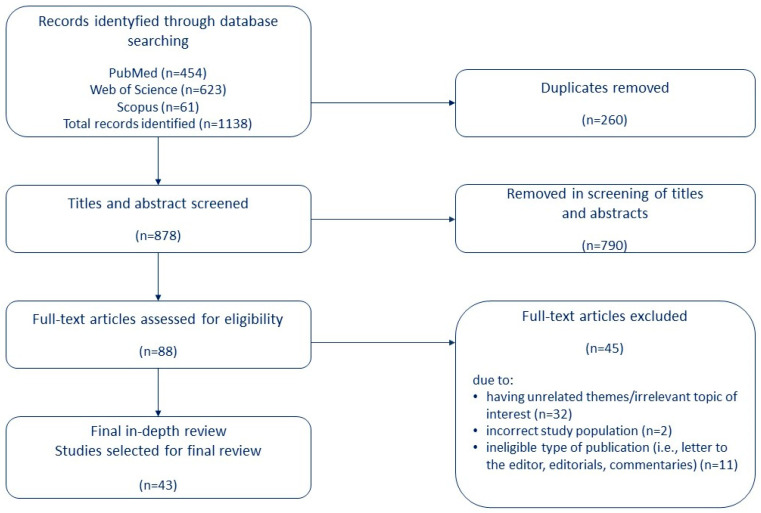
Flow diagram of literature search and study selection.

Three authors independently selected legal acts and papers containing important information on the proposed system solutions to adapt the national health care system in the event of a sudden influx of migrants and/or war refugees. The list of qualified articles and selected legal acts was presented by each of the persons involved in this process to the other co-authors, who decided to include them in the analysis. Detailed information is provided in Figure 1 and Table S1 (Supplementary Materials).

## 3. Results

In our study, the Building Blocks framework was used to evaluate the impact of migration crisis on health system in Poland, as well as to shape the analysis and guide policy recommendations. We analyzed legal acts in force in Poland relating to health care for migrants, key publications of international organizations on the health of migrants and ensuring access to health services, and scientific publications in this area.

Health care for refugees from Ukraine is provided in Poland on the basis of the European Union Council Implementing Decision (EU) 2022/382 of 4 March 2022 and the Act of 12 March 2022 on assistance to Ukrainian citizens in connection with the conflict of armed forces in that country. The aforementioned act grants the right to medical care provided in Poland to citizens of Ukraine, their spouses, and children who came to Poland after 24 February 2022. The Act provides them the opportunity to obtain a number confirming their identity (PESEL), assigned to all Polish citizens, as well as to set up a Trusted Profile and later access to an electronic individual patient account (IKP).

Foreigners from Ukraine can use health care services on the same terms as persons covered by compulsory or voluntary health insurance. These patients can receive reimbursed prescriptions, referrals, or receive necessary vaccinations [20,21]. Third-country nationals and stateless persons who arrived on the territory of the Republic of Poland from 24 February 2022 due to military operations conducted in the territory of Ukraine may obtain the so-called temporary protection for foreigners applying for international protection. This protection is granted on the basis of Council Implementing Decision (EU) 2022/382 [22].

Persons covered by temporary protection may use medical care in Poland to the extent that the right to benefits is vested in persons covered by compulsory or voluntary health insurance, but may only be provided by service providers implementing a civil law contract concluded with the Head of the Office for Foreigners (e.g., in centers for foreigners) [23]. The provisions of Polish law guarantee equal access to medical services for Polish and Ukrainian citizens, and nationality or citizenship cannot be grounds for admitting patients out of turn.

The challenges identified in the analyses for health care in the face of the migration crisis can be translated into practical actions in our country in the following areas:

- Service delivery: selection of model organizational solutions, introduction of innovative solutions, implementation of services in the field of disease prevention including vaccinations, implementation of health programs or health promotion interventions, patient visits to the facility, teleconsultations, ensuring continuity of therapy, e.g., in the case of oncology patients, facilitating access to health services, removing barriers and difficulties in access to health care, strengthening access to information on the rights and rules for using health services (e.g., through preparation of leaflets written in Ukrainian, hotline for patients in Ukrainian, explaining the differences in the functioning of the health care system in the home country and in host countries), and analyzing the demand for health care services among migrants and war refugees [6,7,9,24,25,26,27,28,29,30,31,32,33,34,35,36,37,38,39,40,41,42,43,44,45,46,47];
- Health workforce: conducting an appropriate employment policy ensuring the availability and appropriate qualifications of health care workers, organizing and mobilizing human resources, ensuring awareness-raising on health inequalities related to migrants and war refugees, ensuring the protection of staff working with the patient against infection (e.g., equipping them with appropriate personal protective equipment, building awareness about the lack of vaccinations among children (e.g., against measles) and the possibility of infection in the event of non-compliance with epidemiological regimes, ensuring that the medical staff is vaccinated), being alert for mental health conditions, and help in communicating with patients in Ukrainian (presence of interpreters in medical facilities [6,19,28,29,44,46,48,49,50,51,52,53,54];
- Health information system: providing information resources, including collecting and monitoring the legitimacy of the collected data, collecting data on patients, e.g., their migration status, country of origin for monitoring and evaluation of implemented activities; introducing ICT tools, including, e.g., search engines for medical facilities where staff speak Ukrainian, open access repository for sharing of resources (guidelines, toolkits in a variety of languages), integration of medical systems and exchange of information on health and provided medical services between facilities in a given country and between different countries (including the country of origin of migrants), and preparing reports [19,25,33,43,44,45,55,56];
- Medical products, vaccines, and technologies: providing medical products, vaccinations, and medical technologies of a certain quality and availability; effects of the economic crisis on availability of medicines/economic availability of drugs, and drug reimbursement [57,58,59,60,61];
- Financing and sustainability: financing, including ensuring the financial security of health care entities, determining and analyzing health care costs, organizing and mobilizing financial resources, reducing financial barriers in the access of migrants and war refugees to health services and medicines [6,44,62,63,64,65,66];
- Leadership/governance: acting as a leader in initiating innovative solutions, supervision over the correctness of health care for migrants, regular communication between people involved in providing services both from the medical staff and representatives of other organizations and sectors, cooperation between health care entities, cooperation with authorities supervising health care entities, ongoing cooperation with health care managers, leadership, and management based on flexible action plans appropriate to the changing situation, and building responsive health systems in host communities [6,19,35,36,44,58,67,68,69].

The proposed reorganization of health care system in Poland is based on three strategic objectives. First, it requires quick adaptation of the Polish health care system to help refugees (by granting access to medical care and medicines and implementation of vaccination programs). Secondly, it effectively uses the resources of the Polish health care system. Lastly, it provides public health protection of the Polish citizens accompanied by the elimination of the problems related to the lack of preventive care and by provision of care for chronic patients within the framework of the primary health care.

There are six operational objectives accompanied by organization-related activities. Their summary can be found in Table 2. Proposed solutions presented in our article are community- and primary care-oriented as resources and time needed for thousands of new hospital beds are far beyond our economical and health care capacities.

### 3.1. Preparation of Medical Entities in the Community to Provide Help for Refugees

It is important to secure the minimum number of medical personnel with the appropriate skill mix in a given area. Preferably, there would be lists of no more than 2000 patients per doctor/nurse/midwife, followed by 2500 patients per medical professional. There should be a possibility to mobilize civilian resources (those who hold license to practice the profession but are not currently employed by a medical entity). Doctors from Ukraine should be included in the health care system with a fast supervised employment path. Additionally, primary and ambulatory care, hospitals, pharmacies, as well as logistic organizations such as volunteers, army, police, voluntary fire brigades (OSP), and farmers’ wives’ associations (KGW) could be involved. The strategy is to firstly include those entities who declare their willingness to help. There could be health centers dedicated for Ukrainian refugees, supervised by Polish managers and public officials.

Online training should be delivered to all medical staff on basic Ukrainian language phrases, typical refugee problems (based on the experience gained), new technologies, and integrated care.

The supply chain for medical facilities should be secured. Refugees should have access to running water, means of communication, access to the telecommunications network, means of transport, evacuation routes for the seriously ill and injured, electricity, food, and financial resources for continuous activity, medicines, medical materials and equipment and places to store them, material and technical stock (minimum security for 14–30 days, considering the number of patient declarations of a given primary health care center (PHC), population on the area of PHC activity, and the number of medical entities), infrastructure of the rooms adapted to the requirements of treatment (projects under the Regional Operational Program), and marking of the facilities in accordance with the relevant conventions or concealing the facilities against subversive activities.

### 3.2. Development and Implementation of the Communication System

New systemic organizational solutions should be introduced (data entry, biometrics in all places: border crossing, reception point, registration in municipal and city offices, e.g., personal identification procedures (Universal Electronic System for Registration of the Population (PESEL)), Trusted Profile). To supplement these solutions, patient information cards can be introduced with basic personal information, contact, individual medical care plan (IPOM), health condition summary, and treatments performed during the crisis.

Refuges should be educated on navigating the health system and provided with basic information on prevention, hygiene, and lifestyle. First aid points should be designated. Educational materials on self-care and avoidance of potential risks should be distributed to refugees. For diagnostic procedures, interpreters should be present.

### 3.3. Organization of the Diagnostic Procedures

Diagnosis should take place as early as possible, preferably right after crossing the border or on relocation to a specific place. Basic information on health condition of refugees (vaccinations, allergies, and diseases) and contact details should be collected. Scope of health check-ups for adults and children should be developed. At minimum, those questionnaires should include information on personal and family interviews, contact persons from Ukraine/Polish caretaker, place of shelter, means of transport, allergies, addictions, physical examination, primary prevention (vaccinations) and secondary prevention (cardiovascular and oncology), diagnosis of chronic diseases, chronic medications, action plan, and contact with the Polish PHC coordinator. Screening should be provided in a voluntary, confidential, and non-stigmatizing manner.

Comprehensive visits to primary health care centers including interview, diagnosis of chronic diseases, necessary specialist consultations (past or future), laboratory tests, medications, and performed vaccinations should be documented. There should be screening tests for infectious diseases, as well as list of required vaccinations. Implementation of preventive vaccinations and verification of the vaccination status of refugees is a major challenge for the health care system due to lack of access to existing documentation. In the event of permanent unavailability of vaccination documentation, it is advisable to establish an individual vaccination schedule (in accordance with applicable regulations in Poland). This approach is in line with ECDC recommendations [70].

### 3.4. Organization of Medical Services

In-patient and out-patient care can be coordinated by using data on patients’ health conditions. Services should be obtained on the Polish national Electronic Health Record (her) Platform, called P1 Platform, ultimately providing a free software for users, offering coordinated and integrated basic care. The principle of call back and reverse call should be introduced. This strategy should allow for providing home visits, providing emergency home visits, and online visits. Psychological services to treat trauma should be provided, particularly for Ukrainian children. Lastly, there should be implementation of recommended vaccinations. It is not recommended to vaccinate refugees at border crossing points, but if the risk of transmitting serious disease(s) across the border is considered high in the epidemiological risk assessment, the host country may decide to perform vaccinations based on existing recommendations.

### 3.5. Implementation of Changes in the Management of Medical Entities

P1 platform in cooperation with the County Coordination Center should be used for management (in similar way it was used for COVID-19 cases management). Knowledge gained about the refugee population should be systemically assimilated by coordinating an integrated database. Patient navigation should be introduced for PHC (emergency aid and prevention) and after-hours medical services (NPL) according to the PHC maps. There should be minimal use of hospital information systems (HISs) and paper documentation.

Coordinated care should be implemented. Some of the doctors’ duties should be assigned to nurses and midwives and in the same manner, assignment of nurses’ tasks to other less skilled personnel in the properly arranged pyramid of services and tasks. Functional integration of the teams would be possible thanks to IT systems. These plans require changes in the legal acts (description of tasks and guidelines for the personnel and changes in the remuneration). On-call care should be considered in medical entities, also at night.

### 3.6. Assessment of Changes in the Management of Medical Entities

Reorganization proposal is followed by examples of indicators that can be assigned to individual operational objectives:

1. Number and rate of refugees assisted within the system;
2. Number of patients under constant PHC supervision, annual and two-year participation, especially for chronically ill patients;
3. Number of diagnostic tests;
4. Percentage of pregnant women under medical supervision from the 10th week of pregnancy;
5. Number of obligatory childhood vaccinations and additional COVID-19 vaccinations, hepatitis, pertussis, and other vaccinations;
6. Number of annual health check-ups among underage refugees;
7. Number of annual health check-ups among adult refugees (minimum % participation in preventive screenings for cardiovascular diseases, cytology, mammography).

## 4. Discussion

The war in Ukraine increases the risk for humanitarian and public health emergencies, particularly in countries bordering Ukraine [49]. The appearance of a large group of migrants and war refugees from Ukraine in Poland forced numerous actions to be taken in the field of health policy and legal regulations. The conducted analysis indicates the importance of organizational changes for the functioning of health care in the face of the migration crisis. A multidisciplinary approach to the provision of health care for migrants and war refugees, also in terms of its organization, management, and testing of the effectiveness of interventions, translates into health effects and reducing social inequalities in health.

Although multiple systemic and organizational measures were developed and deployed for Ukrainian refugees in Poland, reviews on the matter show that there are no specific procedures in place which translate to operational tasks for entities in public finance sector and local and reginal levels of government [71]. According to the calculations of the Polish National Cancer Registry in Ukrainian refugees population, 3300 new cases of cancer are anticipated each year, with an overall prevalence of around 52,000 cases [72].

The conflict in Ukraine has highlighted the need for developing such procedures and a comprehensive response package in advance.

In previous studies, the scientific literature and guidance documents developed on topics on health care provision for migrants and refugees have focused on service provision using evidence-based and cost-effective strategies. The strategy proposed by us consists of ways to provide services to immigrants but also securing health care accessibility for Polish residents and the safety of medical staff. This allows for a comprehensive approach to health care reorganization.

In the literature, a lot of attention is provided to providing culturally sensitive health care to migrants to avoid discrimination [10,73]. Most of the proposed solutions focus on separating crisis preparedness education from language and culture training [74]. This can be implemented as continuous support to refugees overcoming barriers in accessing health care, but also as training of health care professionals [75]. The proposed strategy includes both health care professionals’ (HCPs) education and providing information to refugees on health system navigation and disease prevention. It also foresees the presence of interpreters during diagnostic procedures. Countries with highly developed strategies and legislation around health care provision for migrants use a similar approach. In Sweden, civic orientation classes are organized by county councils and include information on health services. The presence of interpreters is mandated through a legislative act (Förvaltningslagen para. 8) and the cost is covered by the government [4]. In Ireland, orientation programs are provided in the Reception Centre for Protection Applicants and Reception Centers for Programme Refugees [4]. Switzerland employs a website with health information for migrants in their native language [76]. In designing community-based strategies for management of non-communicable diseases, best practices include using culturally and linguistically sensitive education, and involvement of migrant communities and outreach approaches through families and peers [73]. What is already provided in Poland for Ukrainian refugees, is free psychological counseling organized by the head/mayor of the municipality where they reside [71,77].

The proposed strategy includes in-depth screening and diagnostic procedures administered preferably at the border. Although this provides a health care system with information needed for successful health care management and delivery of appropriate services, it is important to mention that a recent meta-analysis showed that restrictive entry policies were associated with increased levels of poor mental health (psychological distress, depression, anxiety, and post-traumatic stress disorder), and increased odds of poor self-rated health. The effect was observed with low certainty [78].

An important part of the proposed plan is the systemic assimilation of knowledge about the population through an integrated database. Switzerland’s National Program on Migration and Health addressed immigrant health outcomes through two surveys and studies on mother and child health care, health care for undocumented migrants, patient safety, medical decision making, and the impact of interpreting in the health system [76].

Attention should also be paid to the need to change the understanding of the concept of readiness, resilience, and adequacy in the response of health care systems to crisis situations, e.g., related to the sudden influx of people and the need to meet their health needs. The challenge is each time to maintain consistency between the theoretical models of response and the practice of actions. Formulating new goals, implementing new approaches, and monitoring and evaluating implemented actions to adapt health care to new and changing environmental conditions can significantly contribute to improving the functioning health care for both migrants and the host country population, and strengthen preparedness in responding to future health emergencies.

Multi-sectoral cooperation at various levels of population health management, integration of health policies, consistent communication, and involvement of various health care stakeholders are not only directions of action related to entire health systems, but also to its individual elements, which undoubtedly include protecting the health of migrants and war refugees seeking refuge and safety in other countries.

This study attempts to identify directions for improving health care, which can become the subject of further research to verify their legitimacy. The ability of health care to respond adequately to emerging threats to life and health, including new health needs and different ways of providing health care to all those in need, is a huge challenge in many health care systems around the world.

Refuges and asylum seekers are medically and socially vulnerable. Provision of medical care for them brings not only organizational but also ethical challenges. When making decisions on care organization, prevention, or services available for migrants, policymakers face difficult ethical questions and trade-offs. They must decide to what extent to include migrants in health services (including high-cost therapies) or vaccination programs [79]. Applying ethical principles and frameworks can help policymakers in identifying competing interest and appropriately determining priorities. Health and access to medical care are considered a universal right and they should be provided without discrimination [80]. Policies should rather strive to eliminate health disparities affecting immigrants, refugees, or asylees. Ethical considerations are especially important for allocation of limited resources. Most importantly, decisions should not be based on “social worth, perceived obstacles to treatment, patient contribution to illness, past use of resources, or other non-medical characteristics” [81]. At last, while designing care pathways for refugees, policymakers and health care workers must respect refugees’ right to make their own medical decisions in accordance with their values and preferences [82].

The influx of refugees to Poland in connection with the war in Ukraine revealed the strengths and weaknesses not only of the health care system but also of the entire country. The strength of the public health system is the ability of the public health system to include over 1 million people in the guarantee of access to health services and quick regulations regarding the introduction of medical and medical-related professionals from Ukraine to the labor market.

At the same time, the current situation has revealed institutional weaknesses both at the level of open care (mainly primary health care) and closed care, expressed mainly in limited human and financial resources as well as competence (mainly language and social resources). Additionally, the problem of the Polish system is the disintegrated and ineffective school of medicine which requires a comprehensive reform. Other important deficiencies include an underdeveloped model of psychiatric care (the end of the pilot project, lack of evaluation and unclear announcements regarding the target changes) as well as the lack of effective rules of cooperation between social and health care.

Although our strategy is a comprehensive effort to propose a reorganization scheme for Polish health care system, it is subjected to some limitations. Most importantly, even though proposed activities are aligned with most recent guidelines and best practices on migrant health care, the strategy is merely a brainstorming effort and was not subjected to testing and real life evaluation. Another limitation of our study was that our literature search was limited to papers published in English and we did not identify relevant studies published in other languages.

The implications of the abrupt increase in population for the Polish health system are huge. The system must to evolve into a system suitable for a larger and more diverse population. Reorganization of the system is required to respond to an unavoidable and probably long-lasting increase in the demand for health care services.

The proposed strategy of reorganization of Polish health care system to account for influx of Ukrainian refugees is in line with best practices and guidance from leading health organizations (WHO and ECDC). Framework can be used for planning and service commissioning as well as evaluating quality of delivered services. It also provides a structure for implementation of activities in similar emergency situations in the future.

Experience in taking care of migrants and the domestic population indicate the need for cooperation of representatives of various environments in developing methods of responding to health crises on a global scale and creating a database of good practices using the experience of countries involved in health care for migrants and war refugees.

## 5. Conclusions

The contextual approach to the functioning of health care for migrants and the native population justifies the statement that the migration crisis has proved the need to carry out current and future organizational changes as a response to potential health threats.

The research results show that systemic solutions in the planning and implementing of health care services for migrants and war refugees should cover the following areas of activity:

- Ensuring the availability of human, material, and financial resources as a condition of readiness in responding to emerging health threats;
- Adjusting the allocation and/or reallocation of resources in connection with new/additional needs of health care entities during a sudden, abrupt increase in the number of patients;
- Development of organizational and economic tools (toolkits) for health care entities during the health crisis;
- Cooperation between health care entities and health care stakeholders at the national and local level;
- Creating a database of good practices, using the experience of other health systems and learning from each other.

The proposed strategy of health care reorganization may inform practices and policies that improve the quality of health care and minimize health care inequalities for migrant and war refugees.

## Figures and Tables

**Table 1:** Basic data on health care resources and activity indicators in Poland and the estimated need to increase resources and health services as a consequence of the influx from Ukraine into Poland.

	Average Value 2018–2020		Demand for Additional Population		
	N	Population-Related Indicator	+1 Million	+2 Million	+ 3 Million
Medical staff		per 10,000 population			
physicians	90,890	23.7	2370	4740	7110
dentists	13,595	3.5	354	709	1063
nurses	192,392	50.1	5016	10,033	15,049
midwives	22,992	11.6	599	1199	1798
paramedics	10,503	2.7	274	548	822
Consultations	in thousands	per capita	in thousands	in thousands	in thousands
provided by physicians (in total)	278,734	7.3	7268	14,353	21,803
primary health care	167,251	4.4	4361	8722	13,083
General hospitals	912	per 10,000 population			
beds in total	172,042	44.9	4486	8972	13,457

Source: Statistics Poland [18].

**Table 2:** Operational objectives accompanied by organization-related activities.

Operational Objective	Organization-Related Activities
Preparation of medical entities in the community to provide help for refugees	Securing a minimum number of medical personnel with appropriate skill mix in a given area; Inclusion of doctors and health care personnel from Ukraine in the health system; Online training for all medical staff; Improved awareness of migrant and war refugee health disparities; Ensuring safety for medical personnel; Securing supply chain for medical facilities.
Development and implementation of a communication system	Biometrics, PESEL, and Trusted Profile; Educating refugees in the field of navigating the health system and providing basic information on prevention; Introduction of the patient information card; Providing interpreters at the time of diagnostic procedures; Refugee host families and members of non-govermental organization can assist with navigation of the health system.
Implementation of available digital solutions	Individual patient account (IKP); Web-based patient–professional communication over the patient portals; Social media (for, i.e., health education, health information, and e-health literacy).
Organization of diagnostic services	Diagnosing as early as possible, preferably immediately after crossing the border; Scope of the health check-up for adults/ children; Introduction of comprehensive visits to primary health care centers, screening for infectious diseases; Determination of the required vaccinations.
Organization of medical services	Using data on patients’ health condition to coordinate in-patient and out-patient health care; Health promotion and disease prevention programs for the Ukrainian community in Poland in order to improve the health situation and reduce social inequalities in health; Health education in various settings (educational institutions, workplaces, health care institutions, and social welfare institutions), community-based/multi-lingual health education; The use of medical personnel working in kindergartens/schools (where Ukrainian children attend) and/or workplaces (adult citizens of Ukraine), e.g., school nurses; Coordination and continuity of patient care; Provision of home visits; Providing psychological services to treat trauma.
Implementation of changes in the management system of health care entities	Order management on Polish national Electronic Health Record (EHR) Platform, P1; System assimilation of knowledge about the population; Introduction of the navigation for PHC; Limitation of the use of paper documentation; Implementation of coordinated care; Cooperation between health care entities and health care stakeholders at the national and local level; Creating a database of good practices.

## Data Availability

The data that support the findings of this study are available from the corresponding author upon reasonable request.

## Supplementary Materials

The following supporting information can be downloaded at: https://www.mdpi.com/article/10.3390/ijerph20053831/s1.
