# Supplementary material for: Health Care Organization in Poland in Light of the Refugee Crisis Related to the Military Conflict in Ukraine
Source: Int J Environ Res Public Health. 2023 Feb 21;20(5):3831. doi: 10.3390/ijerph20053831 (PMC10001563; doi:10.3390/ijerph20053831)
Supplement: Supplementary file 1 [file ijerph-20-03831-s001.zip › ijerph-2133080-supplementary.pdf]

Table S1. Overview of included studies (characteristics of articles included in the thematic analysis).

	Full citation	Country	Year of publication	Study design/ method/ Data collection method	Study aims/objectives
1.	Terasaki G, Ahrenholz NC, Haider MZ. Care of Adult Refugees with Chronic Conditions. Med Clin North Am. 2015 Sep;99(5):1039-58. doi: 10.1016/j.mcna.2015.05.006.	USA	2015	Review	This article discussed management strategies for common challenges that arise in the primary care of refugees.
2.	Doocy S, Lyles E, Roberton T, Akhu-Zaheya L, Oweis A, Burnham G. Prevalence and care-seeking for chronic diseases among Syrian refugees in Jordan. BMC Public Health. 2015 Oct 31;15:1097. doi: 10.1186/s12889-015-2429-3.	USA, Jordan	2015	A cross-sectional study	This study aimed to characterize the prevalence of NCDs and better understand issues related to care-seeking for NCDs among Syrian refugees in non-camp settings in Jordan.
3.	van Berlaer G, Bohle Carbonell F, Manantsoa S, de Béthune X, Buyl R, Debacker M, Hubloue I. A refugee camp in the centre of Europe: clinical characteristics of asylum seekers arriving in Brussels. BMJ Open. 2016 Nov 24;6(11):e013963. doi: 10.1136/bmjopen-2016-013963.	Belgium	2016	A cross-sectional study	This study aimed to describe the demographic and clinical characteristics of asylum seekers who arrived in a huddled refugee camp, in the centre of a well-developed country with all medical facilities.
4.	Nnadi C, Etsano A, Uba B, Ohuabunwo C, Melton M, Wa Nganda G, Esapa L, Bolu O, Mahoney F, Vertefeuille J, Wiesen E, Durry E. Approaches to Vaccination Among Populations in Areas of Conflict. J Infect Dis. 2017 Jul 1;216(suppl_1):S368-S372. doi: 10.1093/infdis/jix175.	USA, Nigeria	2017	Review	The aim of this article was to examine key strategic and operational tactics that have led to increased polio vaccination coverage among populations living in diverse conflict settings, including Nigeria, Somalia, and Pakistan, and how these could be applied to reach and vaccinate populations in other settings across the world.

5.	Pavli A, Maltezou H. Health problems of newly arrived migrants and refugees in Europe. J Travel Med. 2017 Jul 1;24(4). doi: 10.1093/jtm/tax016.	Greece.	2017	A systematic review of the scientific literature	The objective of this article was to assess migrants and refugees' health problems, and to recommend appropriate interventions.
6.	Mangrio E, Sjögren Forss K. Refugees' experiences of healthcare in the host country: a scoping review. BMC Health Serv Res. 2017 Dec 8;17(1):814. doi: 10.1186/s12913-017-2731-0.	Sweden	2017	A systematic review of the scientific literature	The aim of this scoping review was to compile research about the experiences that the refugees have with the healthcare systems in their host countries.
7.	Echeverri C, Le Roy J, Worku B, Ventevogel P. Mental health capacity building in refugee primary health care settings in Sub-Saharan Africa: impact, challenges and gaps. Glob Ment Health (Camb). 2018 Aug 28;5:e28. doi: 10.1017/gmh.2018.19.	USA, Belgium, Ethiopia, Switzerland	2018	Intervention	This paper described the results of a process evaluation of a real-life implementation project by an external consultant, one and a half years after starting the programme.
8.	Juárez SP, Honkaniemi H, Dunlavy AC, Aldridge RW, Barreto ML, Katikireddi SV, Rostila M. Effects of non-health-targeted policies on migrant health: a systematic review and meta-analysis. Lancet Glob Health. 2019 Apr;7(4):e420-e435. doi: 10.1016/S2214-109X(18)30560-6.	Sweden, UK, Brazil	2019	A systematic review of the scientific literature and Meta-Analysis	The aim of this systematic review and meta-analysis was to comprehensively examine the effect of non-health-targeted policies on migrant health.
9.	Blake DP. Key Ways to Prevent Infection When There Is No "Building": Aspects for the Field. Surg Infect (Larchmt). 2019 Feb/Mar;20(2):115-118. doi: 10.1089/sur.2018.290.	USA	2019	Review	This manuscript provided a brief historical review of the development of infection control practices and further identifies and outlines several aspects necessary to successful program applications in austere environments.
10.	Chiarenza A, Dauvrin M, Chiesa V, Baatout S, Verrept H. Supporting access to healthcare for refugees and migrants in European countries under particular migratory pressure. BMC Health Serv	Italy, Belgium, England	2019	A mixed method approach (interviews, focus groups, and a literature review)	The aim of this study was to inform the development of a "Resource Package" to support European Union (EU) member states in improving access to healthcare for refugees, asylum seekers and other migrants.

	Res. 2019 Jul 23;19(1):513. doi: 10.1186/s12913-019-4353-1.				
11.	Baykan N, Aslaner MA. The use of emergency department and outpatient clinics by Syrian refugees. J Glob Health. 2019 Dec;9(2):020404. doi: 10.7189/jogh.09.020404.	Turkey	2019	The retrospective study (based on data from the hospital's medical records)	This study aimed to determine which clinics are preferred by Syrian patients and to compare this data to the use of these clinics by other local patients.
12.	Gunst M, Jarman K, Yarwood V, Rokadiya S, Capsaskis L, Orcutt M, Abbara A. Healthcare access for refugees in Greece: Challenges and opportunities. Health Policy. 2019 Sep;123(9):818-824. doi: 10.1016/j.healthpol.2019.06.003.	Australia, UK	2019	Review	The aim of this article was to explore the evolution of the health response for refugees in Greece over the last three years, the challenges faced at different times of the response and the efforts to integrate refugees into Greece's health system.
13.	Tayfur I, Günaydin M, Suner S. Healthcare Service Access and Utilization among Syrian Refugees in Turkey. Ann Glob Health. 2019 Mar 20;85(1):42. doi: 10.5334/aogh.2353.	Turkey	2019	A retrospective observational registry study	This study aimed to illustrate the healthcare services provided to Syrian refugees in Turkey, which is the country hosting the largest number of people displaced in the wake of the civil war in Syria.
14.	Riza E, Kalkman S, Coritsidis A, Koubardas S, Vassiliu S, Lazarou D, Karnaki P, Zota D, Kantzanou M, Psaltopoulou T, Linos A. Community-Based Healthcare for Migrants and Refugees: A Scoping Literature Review of Best Practices. Healthcare (Basel). 2020 Apr 28;8(2):115. doi: 10.3390/healthcare8020115.	Greece, The Netherlands, USA, UK	2020	A systematic review of the scientific literature	The aim of this paper was to identify requirements, prerequisites, and concrete steps to design and implement community-based healthcare models serving migrants and refugees.
15.	Shah S, Munyuzangabo M, Gaffey MF, Kamali M, Jain RP, Als D, Meteke S, Radhakrishnan A, Siddiqui FJ, Ataullahjan A, Bhutta ZA. Delivering non-communicable disease interventions to women and children in conflict settings: a systematic review. BMJ Glob	Canada, Singapore, Pakistan	2020	A systematic review of the scientific literature	The aim of the review was to synthesise the available indexed and grey literature reporting on how NCD interventions are being delivered to these vulnerable populations of women and children, with a specific focus on intervention delivery approaches, barriers and facilitators.

	Health. 2020 Apr;5(Suppl 1):e002047. doi: 10.1136/bmjgh-2019-002047.				
16.	Chua AQ, Tan MMJ, Verma M, Han EKL, Hsu LY, Cook AR, Teo YY, Lee VJ, Legido-Quigley H. Health system resilience in managing the COVID-19 pandemic: lessons from Singapore. BMJ Glob Health. 2020 Sep;5(9):e003317. doi: 10.1136/bmjgh-2020-003317.	Singapore	2020	Review	The aim of this review was to present the response of Singapore to the COVID-19 pandemic based on core dimensions of health system resilience during outbreaks.
17.	Lebano A, Hamed S, Bradby H, Gil-Salmerón A, Durá-Ferrandis E, Garcés-Ferrer J, Azzedine F, Riza E, Karnaki P, Zota D, Linos A. Migrants' and refugees' health status and healthcare in Europe: a scoping literature review. BMC Public Health. 2020 Jun 30;20(1):1039. doi: 10.1186/s12889-020-08749-8.	Sweden, UK, Spain, France, Greece	2020	A systematic review of the scientific literature	The review aimed at identifying what is known about access to healthcare as well as healthcare use of migrants and refugees in the EU member states.
18.	Hasanović M, Šmigalović D, Fazlović M. Migration and Acculturation: What We Can Expect in the Future. Psychiatr Danub. 2020 Oct;32(Suppl 3):386-395.	Bosnia and Herzegovina	2020	Review	This review aimed to identify the biggest challenges for migrant populations within “host” countries.
19.	Rass E, Lokot M, Brown FL, Fuhr DC, Asmar MK, Smith J, McKee M, Orm IB, Yeretzian JS, Roberts B. Participation by conflict-affected and forcibly displaced communities in humanitarian healthcare responses: A systematic review. J Migr Health. 2020 Dec 9;1-2:100026. doi: 10.1016/j.jmh.2020.100026.	UK, Jordan, Lebanon	2020	A systematic review of the scientific literature	The aim of this study was to understand the role of community participation in humanitarian health responses for conflict-affected populations (including forcibly displaced populations) in low- and middle-income countries and the barriers and facilitators to community participation in healthcare responses.
20.	Fuhr DC, Acarturk C, Sijbrandij M, Brown FL, Jordans MJD, Woodward A, McGrath M, Sondorp E, Ventevogel P, Ikkursun Z, El Chammay R, Cuijpers P, Roberts B. Planning the scale up of brief psychological interventions using theory	UK, Turkey, The Netherlands, Switzerland, Lebanon	2020	Intervention	The aim of this study was to unpack pathways for scaling up the World Health Organization’s Problem Management Plus (PM+) and the Early Adolescent Skills for Emotions (EASE) intervention for Syrian refugees.

	of change. BMC Health Serv Res. 2020 Aug 26;20(1):801. doi: 10.1186/s12913-020-05677-6.				
21.	Kiselev N, Pfaltz M, Schick M, Bird M, Pernille H, Sijbrandij M, de Graaff AM, Schnyder U, Morina N. Problems faced by Syrian refugees and asylum seekers in Switzerland. Swiss Med Wkly. 2020 Oct 26;150:w20381. doi: 10.4414/smw.2020.20381.	Switzerland, Denmark, The Netherlands	2020	A cross-sectional study	This study aimed to identify a broad range of problems faced by Syrian refugees and asylum seekers in Switzerland and to close the existing knowledge gap about the perceived problems of this population. More-over, this knowledge is valuable in informing the adaptation of a brief psychological intervention, Problem Management Plus (PM+), in Switzerland.
22.	Garry S, Checchi F. Armed conflict and public health: into the 21st century. J Public Health (Oxf). 2020 Aug 18;42(3):e287-e298. doi: 10.1093/pubmed/fdz095.	UK	2020	A systematic review of the scientific literature	This paper reviewed the effects that conflict has on the health of individuals by synthesising the available literature. It then describes factors that make people and populations especially vulnerable to these effects. The paper then discusses areas for which evidence, policy and/or programmatic emphasis are, in our view, currently insufficient and requiring of greater attention in order to transition humanitarian public health into the next decades of practice.
23.	Kiselev N, Morina N, Schick M, Watzke B, Schnyder U, Pfaltz MC. Barriers to access to outpatient mental health care for refugees and asylum seekers in Switzerland: the therapist's view. BMC Psychiatry. 2020 Jul 17;20(1):378. doi: 10.1186/s12888-020-02783-x.	Switzerland.	2020	A cross-sectional study	The study aimed to assess the challenges and barriers faced by psychiatrists and psychotherapists working in outpatient settings in Switzerland in treating refugees and asylum seekers to determine the potential capacity of this group to provide mental health care.
24.	Miller NP, Ardestani FB, Dini HS, Shafique F, Zunong N. Community health workers in humanitarian settings: Scoping review. J Glob Health. 2020 Dec;10(2):020602. doi: 10.7189/jogh.10.020602.	USA	2020	A systematic review of the scientific literature	The aimed of this study was to synthesize the growing evidence base on health care service delivery through community health workers (CHWs) in humanitarian settings, with the goal of improving delivery of essential services to the most vulnerable populations.
25.	Seyedin H, Rostamian M, Barghi Shirazi F, Adibi Larijani H. Challenges of Providing Health Care in Complex	Iran	2021	A systematic review of the	This systematic review study was conducted to identify the challenges of health-care delivery in complex emergencies.

	Emergencies: A Systematic Review. Disaster Med Public Health Prep. 2021 Nov 2;17:e56. doi: 10.1017/dmp.2021.312.			scientific literature	
26.	Alseed MM, Syed H, Onbasli MC, Yetisen AK, Tasoglu S. Design and Adoption of Low-Cost Point-of-Care Diagnostic Devices: Syrian Case. Micromachines (Basel). 2021 Jul 27;12(8):882. doi: 10.3390/mi12080882.	Turkey	2021	A cross-sectional study	This article aimed to inform healthcare professionals, product developers and the public about Point-of-Care (PoC) devices by surveying the relevant parties including healthcare workers and doctors working in developing countries.
27.	Bowsher G, El Achi N, Augustin K, Meagher K, Ekzayez A, Roberts B, Patel P. eHealth for service delivery in conflict: a narrative review of the application of eHealth technologies in contemporary conflict settings. Health Policy Plan. 2021 Jun 25;36(6):974-981. doi: 10.1093/heapol/czab042.	UK	2021	A systematic review of the scientific literature	This study categorized various forms of eHealth usage in conflict and aims to identify gaps in evidence to make recommendations for further research and practice.
28.	Ansbro É, Homan T, Qasem J, Bil K, Rasoul Tarawneh M, Roberts B, Perel P, Jobanputra K. MSF experiences of providing multidisciplinary primary level NCD care for Syrian refugees and the host population in Jordan: an implementation study guided by the RE-AIM framework. BMC Health Serv Res. 2021 Apr 26;21(1):381. doi: 10.1186/s12913-021-06333-3.	UK, Jordan, The Netherlands	2021	This retrospective mixed methods evaluation of the MSF NCD programme	The aim of this paper was to summarise the full evaluation, which used the RE-AIM implementation framework to examine the Reach; Effectiveness; Adoption and acceptance of the programme; Implementation fidelity, adaptations and costs; and programme Maintenance over time.
29.	Noubani A, Diaconu K, Loffreda G, Saleh S. Readiness to deliver person-focused care in a fragile situation: the case of Mental Health Services in Lebanon. Int J Ment Health Syst. 2021 Mar 2;15(1):21. doi: 10.1186/s13033-021-00446-2.	Lebanon, UK	2021	A qualitative study design (including 15 semi-structured interviews and 2 participatory group model-	This study aimed to determine how healthcare providers offering mental health and psychosocial support service (MHPSS) at primary care levels in Lebanon perceive mental health and the health system's ability to address the rising mental ill-health burden with a view to identify opportunities for strengthening MHPSS service

				building workshops)	implementation geared towards integrated person focused care model.
30.	Grochtdreis T, Röhr S, Jung FU, Nagl M, Renner A, Kersting A, Riedel-Heller SG, König HH, Dams J. Health Care Services Utilization and Health-Related Quality of Life of Syrian Refugees with Post-Traumatic Stress Symptoms in Germany (the Sanadak Trial). Int J Environ Res Public Health. 2021 Mar 25;18(7):3408. doi: 10.3390/ijerph18073408.	Germany	2021	Randomized controlled trial	The aim of this study was to evaluate the economic consequences of post-traumatic stress symptoms (PTSS) with respect to health care services utilization and costs from the health care payers' perspective, and health-related quality of life (HrQo) for Syrian refugees with mild to moderate PTSS without current psychotherapeutic treatment in Germany based on the "Sanadak" trial.
31.	Klas J, Grzywacz A, Kulszo K, Grunwald A, Kluz N, Makaryczew M, Samardakiewicz M. Challenges in the Medical and Psychosocial Care of the Paediatric Refugee-A Systematic Review. Int J Environ Res Public Health. 2022 Aug 26;19(17):10656. doi: 10.3390/ijerph191710656.	Poland	2022	A systematic review of the scientific literature	The aim of this paper was to report on the topic of communication between refugee minors and health care workers and other public actors from countries experiencing a refugee crisis.
32.	Matsangos M, Ziaka L, Exadaktylos AK, Klukowska-Rötzler J, Ziaka M. Health Status of Afghan Refugees in Europe: Policy and Practice Implications for an Optimised Healthcare. Int J Environ Res Public Health. 2022 Jul 27;19(15):9157. doi: 10.3390/ijerph19159157.	Switzerland, Norway	2022	A systematic review of the scientific literature	The aim of this study was to describe the impact of the risk factors specific to locations and living conditions, how these may lead to illness, the most prevalent diseases, the health access of the female population, as well as their neuropsychological overload, and emphasise the need for further research on conflict-induced health status. The paramount aim was to optimise the medical approach and health services for everyone in the world, irrespective of their country of origin and financial background.
33.	Fatyga E, Dzięgielewska-Gęsiak S, Muc-Wierzgoń M. Organization of Medical Assistance in Poland for Ukrainian Citizens During the Russia-Ukraine War. Front Public Health. 2022 Jul	Poland	2022	Review	The aim of this article was to identify characteristics of all activities and types of medical resources that are provided and organized in Poland for refugees from Ukraine territory affected by the war from the first days of the conflict.

	7;10:904588. doi: 10.3389/fpubh.2022.904588.				
34.	Ludvigsson JF, Loboda A. Systematic review of health and disease in Ukrainian children highlights poor child health and challenges for those treating refugees. Acta Paediatr. 2022 Jul;111(7):1341-1353. doi: 10.1111/apa.16370.	Sweden, USA, Ukraine	2022	A systematic review of the scientific literature	The main aims of this review were to describe the Ukrainian healthcare system and build up a picture of the health of Ukrainian children, with a particular focus on diseases and conditions that are more prevalent in their country. We hope that this will provide valuable information for healthcare organisations and professionals who are working with Ukrainian child refugees, as the health profiles of these children will differ from resident paediatric populations.
35.	Khanyk N, Hromovyk B, Levytska O, Agh T, Wettermark B, Kardas P. The impact of the war on maintenance of long-term therapies in Ukraine. Front Pharmacol. 2022 Nov 24;13:1024046. doi: 10.3389/fphar.2022.1024046.	Sweden, Ukraine, Hungary, Poland	2022	Review	This paper described these challenges in more detail, providing a snapshot of the scenario as of early August, 2022 (unless otherwise stated). It also presents various actions that Ukraine has been taking to minimise the effect of these challenges, and to ensure continuation of treatment to patients who require long-term therapies, despite the existing unfavourable conditions.
36.	Lewtak K, Kanecki K, Tyszko P, Goryński P, Bogdan M, Nitsch-Osuch A. Ukraine war refugees - threats and new challenges for healthcare in Poland. J Hosp Infect. 2022 Jul;125:37-43. doi: 10.1016/j.jhin.2022.04.006.	Poland	2022	Review	The aim of this study was to describe threats and challenges to public health related in particular to infectious diseases and to identify the resources of the healthcare system that are necessary to meet the needs of the recent war refugees and the Polish population.
37.	Rzymski P, Falfushynska H, Fal A. Vaccination of Ukrainian Refugees: Need for Urgent Action. Clin Infect Dis. 2022 Sep 29;75(6):1103-1108. doi: 10.1093/cid/ciac276.	Poland, Ukraine	2022	Review	This paper aimed to discuss the potential 5 major vulnerabilities related to infectious diseases (i.e., COVID-19, measles, pertussis, tetanus, and poliomyelitis) and ultimately offers recommendations for countries receiving a high number of refugees from Ukraine.
38.	Wareńczak-Florczak Ż, Urbański B. What challenges does the humanitarian crisis and large number of refugees from Ukraine pose for Polish oncology? Rep Pract Oncol Radiother. 2022 Jul	Poland	2022	Review	This review described and discussed the difficulties faced by cancer treatment centres in Poland in the time of the influx of Ukrainian war refugees into Poland.

	29;27(3):566-570. doi: 10.5603/RPOR.a2022.0051.				
39.	Baatz RK, Ekzayez A, Meagher K, Bowsher G, Patel P. Cross-border strategies for access to healthcare in violent conflict - A scoping review. J Migr Health. 2022 Mar 19;5:100093. doi: 10.1016/j.jmh.2022.100093.	The Netherlands, UK	2022	A systematic review of the scientific literature	This scoping review identified academic literature on cross-border populations to map the current approach to cross-border populations and to propose a research agenda.
40.	Piotrowicz K, Semeniv S, Kupis R, Ryś M, Perera I, Gryglewska B, Gąsowski J. Disease burden in older Ukrainian refugees of war: a synthetic reanalysis of public records data. Lancet Healthy Longev. 2022 Oct;3(10):e667-e673. doi: 10.1016/S2666-7568(22)00187-8.	Poland	2022	Review	The aim of the study was to estimate this burden in older Ukrainian refugees, given the potential financial and logistical impacts on the health-care systems of receiving countries.
41.	Brown GW, Bridge G, Martini J, Um J, Williams OD, Choupe LBT, Rhodes N, Ho ZJM, Chungong S, Kandel N. The role of health systems for health security: a scoping review revealing the need for improved conceptual and practical linkages. Global Health. 2022 May 15;18(1):51. doi: 10.1186/s12992-022-00840-6.	UK, Belgium, Australia, Switzerland	2022	A systematic review of the scientific literature	The review was guided by the following research questions: 1) What is the existing peer reviewed evidence linking health systems to health security?; 2) What are the essential elements and characteristics of a 'strengthened' health system for health security as understood through the heuristic of the WHO Building Blocks?, and; 3) What examples exist to help demonstrate how improvements in health system core components can improve health security?
42.	Greenaway C, Fabreau G, Pottie K. The war in Ukraine and refugee health care: considerations for health care providers in Canada. CMAJ. 2022 Jul 11;194(26):E911-E915. doi: 10.1503/cmaj.220675.	Canada	2022	A systematic review of the scientific literature	This article aimed to outline clinical considerations for health providers caring for people displaced by the war in Ukraine, based on available evidence and guidance, and discuss how Canada can strengthen its measures to provide health care to currently arriving refugees and prepare for future refugee waves.
43.	Püsküllüoğlu M, Grela-Wojewoda A, Szczubiałka G, Zemełka T, Lompart J, Sałek-Zań A, Kopciński T, Pasieka E, Adamczyk A, Mucha-Małecka A, Kłęk S, Ryś J, Ziobro M. Cancer care for	Poland	2022 (early access date)	A cross-sectional analysis (based on data from the	The aim of this study was:(1) to explore the profile of cancer patients among the refugees; the time of the decision to flee the war-torn country and transfer cancer treatment to another country in the case of Ukrainian cancer patients; (2) to define challenges connected with

	Ukrainian refugees during the first 6 weeks of 2022 Russian invasion - An experience of a cancer reference centre in Poland. Eur J Cancer. 2023 Jan;178:234-242. doi: 10.1016/j.ejca.2022.10.005.			hospital's medical records)	the unexpected inflow of cancer refugee patients from one European country to another; (3) to share what local solutions were introduced to manage the growing number of refugees requiring oncological support.
